# Molecularly Defined Circuitry Reveals Input-Output Segregation in Deep Layers of the Medial Entorhinal Cortex

**DOI:** 10.1016/j.neuron.2015.10.041

**Published:** 2015-12-02

**Authors:** Gülşen Sürmeli, Daniel Cosmin Marcu, Christina McClure, Derek L.F. Garden, Hugh Pastoll, Matthew F. Nolan

**Affiliations:** 1 Centre for Integrative Physiology, University of Edinburgh, Hugh Robson Building, Edinburgh, EH8 9XD, UK

## Abstract

Deep layers of the medial entorhinal cortex are considered to relay signals from the hippocampus to other brain structures, but pathways for routing of signals to and from the deep layers are not well established. Delineating these pathways is important for a circuit level understanding of spatial cognition and memory. We find that neurons in layers 5a and 5b have distinct molecular identities, defined by the transcription factors Etv1 and Ctip2, and divergent targets, with extensive intratelencephalic projections originating in layer 5a, but not 5b. This segregation of outputs is mirrored by the organization of glutamatergic input from stellate cells in layer 2 and from the hippocampus, with both preferentially targeting layer 5b over 5a. Our results suggest a molecular and anatomical organization of input-output computations in deep layers of the MEC, reveal precise translaminar microcircuitry, and identify molecularly defined pathways for spatial signals to influence computation in deep layers.

## Highlights

• The transcription factors Etv1 and Ctip2 distinguish entorhinal layers 5a and 5b
• Layer 5a has extensive intratelencephalic projections, but layer 5b does not
• Terminals of layer 2 stellate, but not pyramidal cells, are enriched in deep layers
• Hippocampal and stellate cell inputs preferentially target layer 5b neurons

## Teaser

Sürmeli et al. demonstrate that input and output functions of the deep layers of the medial entorhinal cortex are divided between two cell populations that are distinguished by their molecular identity, lamina position, translaminar and hippocampal inputs, and telencephalic targets.

## Introduction

Spatial cognition and episodic memory rely on signal processing by the medial entorhinal cortex (MEC), which is believed to function both as a relay of hippocampal outputs to other brain structures and as a major source of cortical input to the hippocampus (Burwell, 2000, van Strien et al., 2009). These separate functions are attributed, respectively, to the deep and superficial layers of the MEC (Burwell and Amaral, 1998, van Strien et al., 2009). Nevertheless, the organization of inputs to and outputs from neurons in the deep layers is not well understood. The identity of neurons receiving hippocampal input is not clear, and the possibility that spatially rich signals from superficial layers of the MEC can influence deeper layers directly, rather than via the hippocampus, has not been investigated experimentally.

The view that deep layers of the MEC relay hippocampal signals to the neocortex is based on two sets of observations. First, axons from hippocampal neurons, and short latency electrical responses following stimulation of the hippocampus, are found in deep layers of the MEC (Kloosterman et al., 2003a, Kloosterman et al., 2003b). Second, neurons in deep layers are labeled by retrograde tracers injected into telencephalic areas, including the neocortex, striatum, and amygdala (Agster and Burwell, 2009, Insausti et al., 1997, Meredith et al., 1990, Swanson and Köhler, 1986). These findings are consistent with a view of deep layer circuits in which hippocampal inputs synapse with neurons that relay their signals directly to telencephalic targets. However, whether the organization of circuitry in deep layers of the MEC is sufficient to support processing beyond that required simply to relay signals is not clear.

The direction of flow of information between superficial and deep layers is of potential importance to theories of spatial cognition and memory, as the high density of grid and border cells in superficial layers of the MEC is believed to be critical for path integration-based estimation of location (Hafting et al., 2005, McNaughton et al., 2006, Moser et al., 2008, Sargolini et al., 2006, Tang et al., 2014). In contrast, deep layers of the MEC contain a high density of cells with activity modulated by head direction and a much lower density of cells with grid-like spatial firing fields (Hafting et al., 2005, Sargolini et al., 2006). Therefore, if deep layers do not receive input from layer 2 (L2), then the grid firing patterns of neurons in deep layers must be generated independently from grid firing in L2, either within deep layers or by inheritance from the pre- or parasubiculum (Boccara et al., 2010, Canto et al., 2012). Conversely, direct connections from L2 could provide a substrate for grid and other spatial representations in deep layers to be inherited from or controlled by L2. Such projections could also provide a path for L2 to influence other brain regions via projection neurons found in the deep layers (van Strien et al., 2009). Distinguishing these possibilities requires experiments that establish whether L2 cells synapse with neurons in deep layers and that determine the identity of any neurons that receive such inputs.

In model systems, principles for connectivity have been established based on the location and molecular identity of presynaptic and postsynaptic neurons (Arber, 2012, Kolodkin and Tessier-Lavigne, 2011, Sürmeli et al., 2011). In L2 of the MEC, there are two major principal cell populations. L2 stellate cells (L2SCs) express the protein reelin and project to the dentate gyrus and CA3 regions of the hippocampus, whereas L2 pyramidal cells (L2PCs) express calbindin and project to CA1 (Kitamura et al., 2014, Varga et al., 2010). In deep layers of the MEC, cells have diverse morphological and electrophysiological characteristics, but sub-laminar organizing principles are unclear (Canto et al., 2008, Gloveli et al., 2001, Hamam et al., 2000). Recently, we found that gene expression patterns delineate layers 5a, 5b, and 6 (Ramsden et al., 2015). However, in contrast to L2, it is not clear if this molecular organization reflects more general principles for organization of connectivity. For example, if projections from L2 to deep layers exist, then it would be important to know whether the specificity of synaptic connections follows a logic that reflects the molecular identity, location, or projections of neurons in the deep layers.

In this study, we use genetic, anatomical, and electrophysiological approaches to establish principles for organization and connectivity of deep layers of the MEC. We show that L5a is a major extra-hippocampal output center of the MEC and is distinguished by differential expression of the transcription factor Etv1 (ETS variant 1). In contrast, we are unable to identify intratelencephalic (IT) projections of L5b, which we find is identified by expression of the transcription factor COUP-TF interacting protein 2 (Ctip2) and may in part project to the thalamus. Utilizing two transgenic mouse lines that give genetic access to L2SCs and L2PCs, we find that the striking differences in efferent targets of L5 neurons are paralleled by specificity of projections from neurons in L2. Thus, L2SCs selectively contact principal cells in L5b while avoiding principal cells in L5a. In contrast, L2PCs appear to make relatively few synaptic contacts with neurons in deep layers. We find that output from the CA1 and subicular region of the hippocampus also preferentially targets layer 5b over 5a. Together, our data define a molecular framework for addressing connectivity of MEC layers, establish an anatomical substrate for spatial representations in L2 to directly influence computation in deep layers of MEC, show that the primary targets of hippocampal inputs have few if any IT projections, and suggest an anatomical segregation of input to and output from deep layers of the MEC.

## Results

### Layers 5a and 5b Have Distinct Molecular Identities and Projection Targets

**Figure 1:**
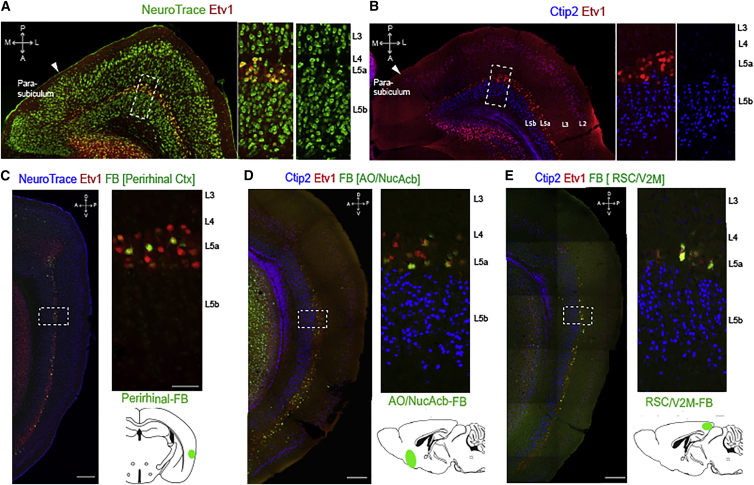
Molecular and Projection Identity Delineate Sub-Layers of Deep MEC (A) Horizontal brain section showing Etv1 immunolabeling (red) in a thin layer of superficial L5 cells (L5a). Neurons are counterstained with NeuroTrace (green). Inset (boxed area) shows the arrangement of cell bodies in L3-L5b. Large cell bodies of layer 5a neurons are underneath the cell free L4 zone and have a scattered organization, whereas L5b contains densely packed neurons with smaller cell bodies. (B) Horizontal section showing Etv1 (red) and Ctip2 (blue) immunolabeling of L5a and L5b, respectively. Inset (boxed area) shows at higher magnification the arrangement of Etv1 and Ctip2 positive zones within layer 5. (C–E) Sagittal sections showing retrograde labeling in the MEC following fast blue ([FB], green) injections into the perirhinal cortex (C), anterior olfactory area (AO) and nucleus accumbens (NucAcb) (D), and retrosplenial cortex (RSC) and secondary visual area (V2M) (E). Projection neurons are located within the zone labeled by Etv1 (red) but are excluded from the adjacent zone labeled by Ctip2 (blue). Diagrams show approximate injection location and coverage. Scale bars are 250 μm for main panels and 50 μm for insets. A: anterior, P: posterior, M: medial, L: lateral, D: dorsal, V: ventral.

Before investigating connections from and to deep layers of the MEC, we first established ways of unambiguously identifying neuronal populations in deep layers using molecular markers. We recently found expression of groups of genes in precise layer specific patterns suggesting that L5 is divided into two molecularly distinct sublayers (L5a and L5b) (Ramsden et al., 2015). We therefore investigated further the pattern of expression of two transcription factors Etv1 (also called Er81) and Ctip2 (also called Bcl11b), which have been used to mark two intermingled subpopulations of neurons in L5 of the neocortex (Chen et al., 2008, Lickiss et al., 2012, Yoneshima et al., 2006). Immunolabeling of MEC using an antibody against Etv1 marked neurons in a narrow zone adjacent to lamina dissecans and corresponding to L5a (n = 15 mice, fraction of labeled neurons 66.8% ± 3.0%, 429/642 cells, n = 3 mice) (Figure 1A). Ctip2 immunolabeling on the other hand marked neurons in a broader and deeper region corresponding to L5b (fraction of labeled neurons 85.8% ± 1.0%, 3,627/4,149 cells, n = 3 mice) (Figure 1B). The cells in L5b have smaller and more uniform soma size compared to the nearby cells in L5a (L5a: 17.7 ± 0.5 μm; L5b: 12.9 ± 0.4 μm n = 3 mice, 120 cells from each layer, paired t test p = 5.1 × 10^−4^) and were relatively densely packed compared to the more scattered L5a neurons (cell body packing density L5a: 1,627.9 ± 149.5 cells/mm^2^ L5b: 3,611.9 ± 277.4 cells/mm^2^, n = 3 mice, paired t test p = 6.6 × 10^−3^). The absence of overlap in expression of the two transcription factors continues across the full dorsoventral and mediolateral extent of the MEC. In contrast, in nearby perirhinal cortex populations of cells expressing Etv1 and Ctip2 intermingle (Figure S1A). These data reinforce the conclusion that deep layers of MEC can be distinguished on the basis of gene expression (Ramsden et al., 2015, Stoya et al., 2014), identify Etv1 and Ctip2 as specific markers of L5a and L5b within the MEC, and suggest that principles for organization of molecularly defined cells in deep layers of MEC differ from neocortical regions.

While differences in the molecular make-up of neuronal populations often correlate with specific axonal projection targets (Greig et al., 2013, Kitamura et al., 2014, Varga et al., 2010), it is not known if the molecular identity or location of neurons in deep layers of MEC map onto their connectivity. To address this, we injected retrograde tracers into a number of cortical and subcortical structures previously shown to receive input from the deep layers of the MEC (Agster and Burwell, 2009, Insausti et al., 1997, Meredith et al., 1990, Swanson and Köhler, 1986). When we demarcated layer 5a and 5b either by cell morphology or by transcription factor expression, we observed retrogradely labeled neurons in layer 5a following injections into perirhinal cortex (PRh) (Figure 1C), nucleus accumbens (NucAcb) and adjacent anterior olfactory area (AO) (Figure 1D), retrosplenial cortex (RSC) and adjacent secondary visual cortex (V2M) (Figure 1E), amygdala, primary visual cortex, cingulate cortex, and the hippocampus (data not shown). We did not find evidence for projections from layer 5b to any of these structures but did observe sparse labeling of L5b neurons in medial sections of MEC following injections into anterior and lateral thalamic nuclei (Figures S1B and S1C). Thus, layer 5 of the MEC is divided into two distinct cell populations with distinct molecular profiles and strikingly different projection targets. L5a appears to be a major output layer of the MEC with projections to diverse cortical and subcortical structures. In contrast, neurons in L5b have distinct connectivity, with few detectable long-range projections, suggesting they may act locally rather than on distant brain regions.

### Connectivity from Superficial to Deep Layers of the MEC Depends on Cell Identity and Target Location

**Figure 2:**
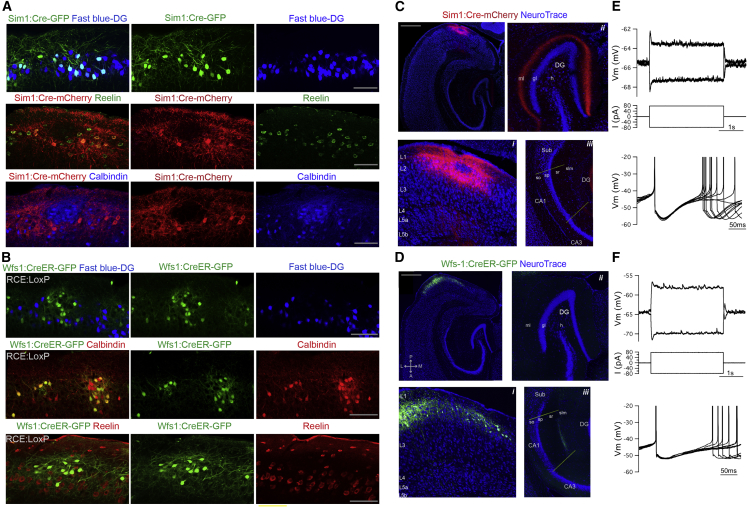
*Sim1*:*Cre* and *Wfs1*:*CreER* Mice Give Genetic Access to L2 Stellate and Pyramidal Cells, Respectively (A) Reporter gene expression (GFP or mCherry) in L2 of the MEC of Sim1Cre mice following injections of AAV-FLEX-GFP (Sim1:Cre-GFP, green) and AAV-FLEX-rev-ChR2-mCherry (Sim1:Cre-mCherry, red), labels cells that are also retrogradely labeled from the dentate gyrus (upper row) and by immunostaining for reelin (middle row) but not calbindin (lower row). First column is a composite of the images in the second and third columns. Note the exclusion of mCherry-positive cell bodies and processes where a calbindin positive cell island is located. For quantification see Figure S2B. (B) Reporter gene expression (GFP) in *Wfs1*:*CreER*; *RCE*:*LoxP* mice (Wfs1:CreER-GFP, green) labels cells in MEC L2 that are not retrogradely labeled from the dentate gyrus (upper) or immunolabeled for reelin (middle) but are positive for calbindin (lower). For quantification see Figure S2B. (C and D) Horizontal brain sections showing Sim1:Cre-mCherry cells (C) and Wfs1:CreER-GFP cells (D). Insets show the layer 2 restriction of the injections (i) and presence or absence of axonal projections in the DG (ii) and CA1 (iii). Images in (ii) and (iii) are post-processed to increase pixel brightness in order to reveal axonal labeling. (E and F) Example of membrane potential responses (upper) to current steps (middle) and overlaid consecutive threshold action potentials (lower), recorded from visually identified Sim1:Cre-mCherry (E) and Wfs1:CreER-eGFP (F) neurons. For quantification of data, see text and Figure S2. Scale bars for (A) and (B) are 100 μm and for (C) and (D) are 500 μm. Abbreviations: DG, dentate gyrus; ml, molecular layer; gl, granular layer; h, hilus; Sub, subiculum; so, stratum oriens; sp, stratum pyramidale; sr, stratum radiens; slm, stratum lacunosum moleculare.

To be able to test whether deep layers receive input from neurons in L2, we first identified transgenic mouse lines giving genetic access to the stellate and pyramidal neurons in L2. To achieve L2-specific expression, we developed an injection strategy that precisely targeted L2 within approximately the dorsal half of the MEC (Figures 2 and S2A; Experimental Procedures). Following this strategy, we investigated Cre recombinase activity in a transgenic mouse line where Cre expression is controlled by the Single minded homolog-1 (Sim1) promoter (*Sim1*:*Cre* mice). In these mice, we injected into the MEC, Cre-dependent adeno-associated virus encoding either GFP (AAV-FLEX-GFP) (Murray et al., 2011) or ChR2(H134R)-mCherry (AAV-FLEX-rev-ChR2-mCherry) (Atasoy et al., 2008), and injected the retrograde tracer fast blue into the dentate gyrus. Cells expressing the reporter gene were positive for reelin and projected to the dentate gyrus but were not positive for calbindin (Figures 2A, S2B, and S2C). We also investigated transgenic mice expressing tamoxifen inducible Cre recombinase under the control of the Wolfram syndrome 1 homolog (Wfs1) promoter (*Wfs1*:*CreER* mice). When these mice were injected with AAV-FLEX-GFP, or crossed with the *RCE*:*loxP* reporter line (Miyoshi et al., 2010), neurons expressing GFP were positive for calbindin, although only a subset of calbindin-positive neurons expressed the reporter gene (Figures 2B and S2B). Neurons expressing GFP were not retrogradely labeled from the dentate gyrus and were negative for reelin (Figure 2B). A lack of overlap between reporter gene expression and parvalbumin indicates neither line drives Cre expression in parvalbumin interneurons (Figures S2B and S2C). Thus, *Sim1*:*Cre* mice give selective genetic access to a L2 cell population that is positive for reelin and projects to the dentate gyrus, while *Wfs1*:*CreER* mice give access to a cell population that is positive for calbindin and does not project to the dentate gyrus.

To further evaluate the specificity of cell labeling obtained with the *Sim1*:*Cre* and *Wfs1*:*CreER* mice, we asked if Cre-expressing neurons recapitulate the distinct hippocampal projections and intrinsic electrophysiological properties of L2SCs and L2PCs (Kitamura et al., 2014, Varga et al., 2010). In *Sim1*:*Cre* mice, anterograde axonal labeling was observed in the inner molecular layer of dentate gyrus and in stratum lacunosum moleculare of CA3 but was absent from CA1 and other regions (n = 4 mice) (Figure 2C). This is consistent with the projection patterns of L2SCs (Dolorfo and Amaral, 1998, Steward and Scoville, 1976). In contrast, in *Wfs1*:*CreER* mice axons were detected only in CA1 within the hippocampus at the border of stratum radiatum and stratum lacunosum moleculare (Figure 2D; n = 3 mice). This is consistent with observations from another Wfs1:Cre driver line (Kitamura et al., 2014). Whole-cell patch-clamp recordings in adult brain slices revealed that fluorescently labeled neurons in *Sim1*:*Cre* and *Wfs1*:*Cre* mice had intrinsic electrophysiological properties corresponding to those of L2SCs and L2PCs, respectively (Figures 2E and 2F) (cf. Kitamura et al., 2014, Pastoll et al., 2013). Thus, neurons labeled in *Sim1*:*Cre* and *Wfs1*:*Cre* mice differed significantly in their time constant and sag coefficient (time constant, p = 6.1 × 10^−5^; sag coefficient, p = 6 × 10^−3^, unpaired t test) (Figure S2D) and had morphology similar to previously described stellate and pyramidal cell populations (Figure S2E) (Klink and Alonso, 1997, Tang et al., 2014). Together, these data indicate that *Sim1*:*Cre* and *Wfs1*:*CreER* mice give genetic access to neurons with characteristic features of L2SCs and L2PCs.

**Figure 3:**
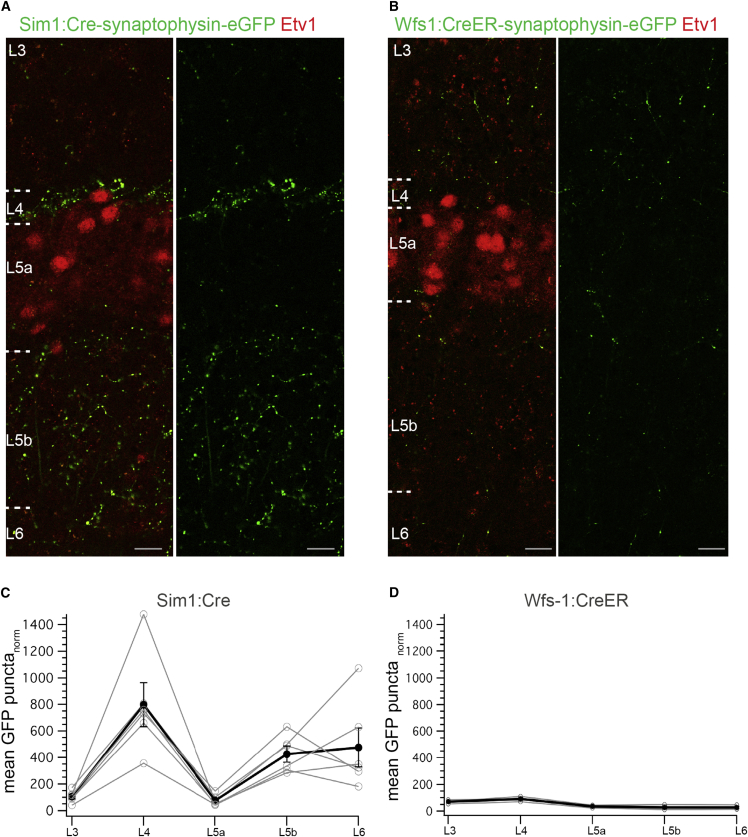
Differences in the Distribution Patterns of L2SC and L2PC Axon Terminals in Deep Layers (A and B) Putative synaptic terminals labeled in deep layers following expression of synaptophysin-eGFP in L2SCs (A) and L2PCs (B) (see Figure S3 for experimental design). Scale bars: 20 μm. (C and D) Normalized density of synaptic terminals plotted as a function of layer. Black line shows the average values for three mice per genotype (mean ± SEM). Note the difference in the overall density of synaptic terminals in *Sim1*:*Cre* versus *Wfs1*:*CreER* mice (Sim1:Cre: 2,190 ± 763 and Wfs1:CreER: 254 ± 36 terminals/mm^2^/number of infected cells in L2 ROI, n = 3 mice per genotype). Gray lines correspond to average values from individual mice. Puncta counts were normalized to the area of the region of interest used for the measurements and to the number of labeled layer 2 cells (See Experimental Procedures and Figure S3).

Having established that neurons in L5a and L5b have distinct molecular identities and projection targets, and that L2SCs and L2PCs can be specifically targeted with Cre driver mouse lines, we were able to ask if either population of deep layer neurons receives inputs from L2. To map the putative synaptic terminals of L2SCs and L2PCs, we injected Cre-dependent AAVs expressing an eGFP tagged form of synaptophysin (AAV-FLEX-synaptophysin-eGFP) into L2 of *Sim1*:*Cre* (n = 6) and *Wfs1*:*CreER* (n = 3) mice (Figure S3). As expected, terminals of L2SCs were found in the middle molecular layer of the dentate gyrus, and terminals of L2PCS were observed in stratum radiatum of CA1 (Figure S3). Within the MEC, abundant axon terminals of L2SCs were differentially distributed across the deep layers (p = 5.7 × 10^−5^, ANOVA) (Figures 3A and 3C). Strikingly, the density of terminals in L5b was >5-fold greater than in L5a (p = 0.0004, paired t test). In contrast to the overall high density of axonal terminals from SCs, expression of synaptophysin-eGFP in L2PCs, while clearly labeling projections to the CA1 region of the hippocampus (Figure S3), revealed far fewer putative synapses in layers 3–6 (p = 6.9 × 10^−6^, ANOVA) (Figures 3B and 3D). When normalized to the number of infected neurons in L2, we found a >10-fold enrichment of terminals labeled in L5b of *Sim1*:*Cre* compared to *Wfs1*:*Cre* mice (adjusted p = 0.004, unpaired t test). Thus, deep layers of the MEC receive inputs from superficial layers that are organized according to the molecular identity of the presynaptic neurons and postsynaptic target zones. Whereas L2PCs make relatively few synaptic contacts in deeper layers, terminals of L2SCs are abundant and are topographically organized. These terminals are enriched in L5b but are excluded from adjacent L5a, suggesting selectivity in the functional connectivity from L2SCs to deep layers of the MEC.

### Monosynaptic Connections from Layer 2 Stellate Cells Selectively Excite Neurons in Layer 5b

To test whether the compartmentalized distribution of putative synaptic terminals reflects targeting to specific neuronal populations, we examined responses of neurons in each layer to activation of L2SCs. To selectively activate L2SCs, we injected AAV-FLEX-rev-ChR2-mCherry into L2 of the MEC of *Sim1*:*Cre* mice. We then tested for postsynaptic responses to light activation of L2SCs using whole-cell recordings from neurons in sagittal and horizontal brain slices containing the MEC. We focused on putative principal cells (n = 138 neurons, 40 mice) (see Experimental Procedures) and excluded less frequently encountered putative interneurons from our analysis (n = 16 neurons). We obtained similar results from experiments using sagittal and horizontal brain slices, and therefore data were pooled (Figure S4A).

**Figure 4:**
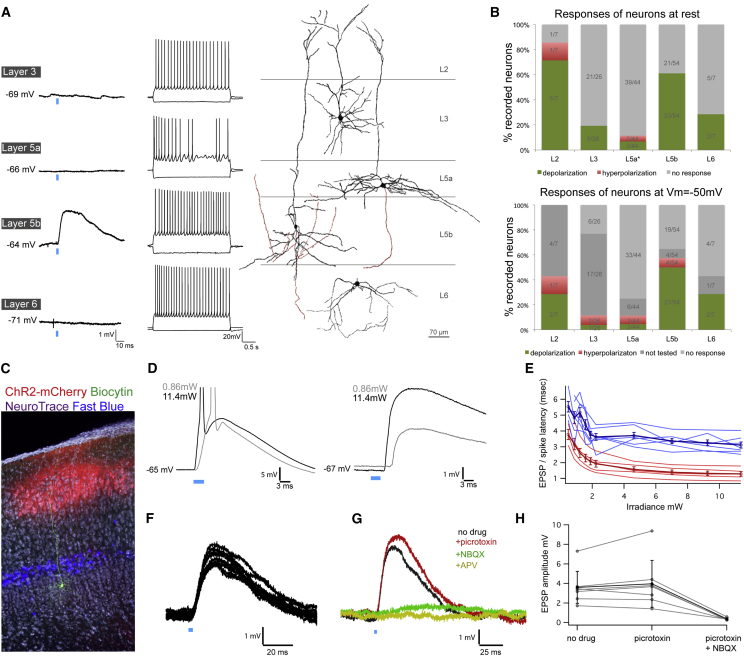
Glutamatergic Projections from L2SCs Selectively Target Neurons in L5b (A) Examples of responses of neurons in each layer to activation of L2SCs (left). Photostimulation (blue bar) of L2SCs evoked PSPs in many L5b principal cells while cells in layers 3, 5a, and 6 were typically not responsive. For each cell, its response to injected current steps (middle) and its morphology (right) are also shown. Dendrites and axons are colored in black and red, respectively. (B) Proportion of responses from cells in each layer following photostimulation of L2SCs at resting membrane potential (−70 ± 0.4 mV) (upper) and when the membrane potential was adjusted to −50 mV with current injections (lower). Green, red, and gray shaded segments, respectively, indicate the percentage of cells in which the membrane potential depolarizes, hyperpolarizes, or does not change. L5a data includes 17 retrogradely labeled cells. L2 data are for non-stellate pyramidal cells. (C) Example of an L5b neuron filled with biocytin (green) for which photostimulation of L2SCs expressing ChR2-mCherry (red) evoked EPSPs. Neurons back-labeled from the nucleus accumbens highlight the location of L5a. NeuroTrace is used as a counterstain (purple). (D) Example of spikes recorded from a ChR2-mCherry expressing L2SC (left) and EPSPs recorded from an L5b neuron (right) during low (0.86 mW, gray trace) and high-intensity (11.4 mW, black trace) light stimulation. (E) The mean latencies of the EPSPs/spikes of seven responding L5b neurons (blue) and five Sim1:Cre-ChR2-mCherry neurons (red) are plotted as a function of light intensity. Thicker lines indicate the population average (mean ± SEM). (F) Examples of ten consecutive responses of an L5b neuron illustrate the short and invariant latency of PSPs. (G) Effects of pharmacological blockers on PSPs recorded from an L5b neuron. (H) EPSP amplitudes before and during application of the indicated pharmacological agents. Gray points are individual cells and black points are the population average (mean ± SEM).

We find that L2SCs preferentially target principal neurons with cell bodies in L5b. Thus, when recording at a cell’s resting membrane potential, activation of L2SCs evoked depolarizing PSPs in the majority of L5b neurons (33/54; mean response amplitude = 1.75 ± 0.26 mV) (Figures 4A, 4B, and S4), but in few neurons in L5a (3/27, mean response amplitude = 1.46 ± 0.4 mV) or L3 (5/26, mean response amplitude = 1.52 ± 0.4 mV) (Figures 4A and 4B). Activation of L2SCs also evoked PSPs in L2PCs and in principal cells in L6 (Figures 4A and 4B). To verify the absence of input from L2SCs to projection neurons in L5a, we recorded from a further 17 L5a neurons identified following injection of retrograde tracer into either the Prh, RSC/V2M, or AO/NucAcb (n = 5, 6, and 6 cells, respectively). These identified projection neurons also failed to respond to activation of L2SCs. Together, these data indicate that neurons in L5b receive functional inputs from L2SCs, whereas projection neurons in L5a do not.

**Figure 5:**
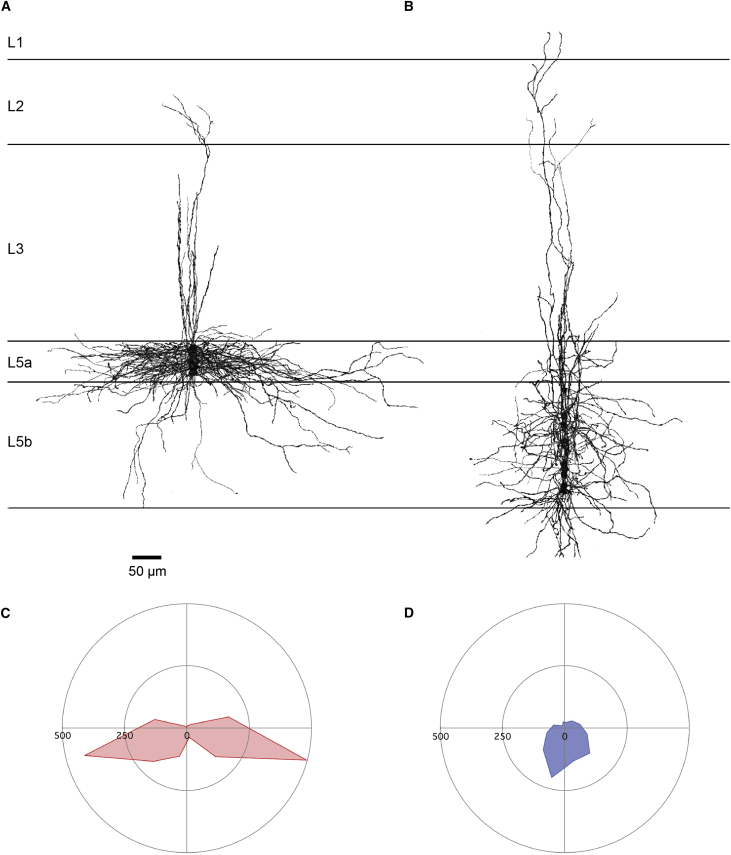
Distinct Dendritic Organization of L5a and L5b Neurons (A and B) Superimposition of reconstructions of ten non-responsive L5a (A) and ten responsive L5b neurons (B). Note the restricted spread of the basal dendrites into adjacent layers. (C and D) Wedge plots of mean total dendritic length of neurons in L5a (n = 10) (C) and L5b (n = 10) (D). The proportion of total dendritic length found in the wedges parallel to the layer border was greater for neurons from L5a compared to L5b (adjusted p = 0.038, t test).

Morphological analysis of neurons reconstructed after recording shows that the responding neurons from L5b were mostly pyramidal in shape with small cell bodies and basal dendrites primarily restricted to L5b (Figures 4A, 4C, 5A, and 5B). Consistent with the synaptic responses being mediated by the terminals of L2SCs found in L5b, a subset of responding L5b neurons had dendrites restricted to layer 5b (n = 7/17). Responding and non-responding neurons in L5b could not be distinguished on the basis of their cell body surface area (L5b non-responding: 544 ± 37 μm^2^ n = 4; L5b responding: 550 ± 44 μm^2^, n = 6, p = 0.9 paired t test) or number of primary dendrites (non-responding: 3 ± 0.7 n = 4; responding: 4.1 ± 0.5, n = 6, p = 0.2 paired t test). The non-responding neurons in L5a had larger cell body surface areas than responding neurons in L5b (L5a: 831 ± 81 μm^2^, n = 9 cells, p = 0.02). Strikingly, alignment of reconstructions of responding cells in L5b indicates that their basal dendrites extend in all directions within layer 5b (Figure 5). In contrast, when we align reconstructions of non-responding cells in L5a, we find that their basal dendrites primarily extend in a medial-lateral orientation and are mostly restricted to L5a (Figure 5). The basal dendrites of L5a neurons had a greater overall length (5a: 2,000.2 ± 127.0 μm, 5b: 1,113.5 ± 137.2 μm, p = 0.0002, t test) and a greater fraction of their basal dendritic tree aligned with the border between L5a and L5b (Figures 5C and 5D). Thus, the dendritic organization of neurons responding to inputs from L2SCs maps closely onto the differential distribution of synaptic terminals of L2SCs in the deep layers.

Are responses of L5b neurons to activation of L2SCs consistent with direct glutamatergic inputs? Light pulses evoked action potentials in L2SCs with short latency and synaptic potentials in L5b neurons with an additional delay of approximately 2 ms (Figures 4D and 4E), which is comparable to monosynaptic local excitatory connections in other cortical circuits (cf. Lefort et al., 2009, Markram et al., 1997). Several further properties of the synaptic responses were also consistent with monosynaptic connectivity: latencies of spiking responses of L2SCs and synaptic responses of L5b principal neurons became shorter as the stimulus intensity was increased, but the relative latency was independent of stimulus intensity (Figure 4E); latencies were relatively invariant from trial to trial (mean SD of the synaptic response latency = 0.39 ± 0.06 at maximal stimulus intensity, n = 11 neurons) (Figures 4F and S4); during repetitive stimulation the EPSP latency, relative to that of action potential firing by SCs, and its variability, were independent of the response’s position within a train (Figure S4D); the probability of evoking a synaptic response as a function of light intensity is similar to the probability of evoking action potential firing by SCs (Figure S4H). EPSPs evoked in L5b neurons were maintained during block of GABA receptors (n = 7/7) and were abolished by the iGluR antagonist NBQX (n = 6/6), indicating that the connections are glutamatergic (Figures 4G and 4H). Consistent with this interpretation, during injection of postsynaptic current to depolarize the membrane potential above the GABA reversal potential, activation of L2SC inputs continues to evoke EPSPs in the majority of tested L5b neurons (n = 27/31). In the remaining L5b neurons (n = 4/31) and in a larger proportion of responding neurons in layers 3 and 5a, responses reversed polarity, suggesting they were mediated by GABAergic synapses (Figure 4B, lower panel). Together, these data indicate that responses of L5b principal neurons to activation of L2SCs are mediated by monosynaptic activation of glutamatergic synapses.

### Hippocampal Projections Preferentially Target Neurons in Layer 5b

**Figure 6:**
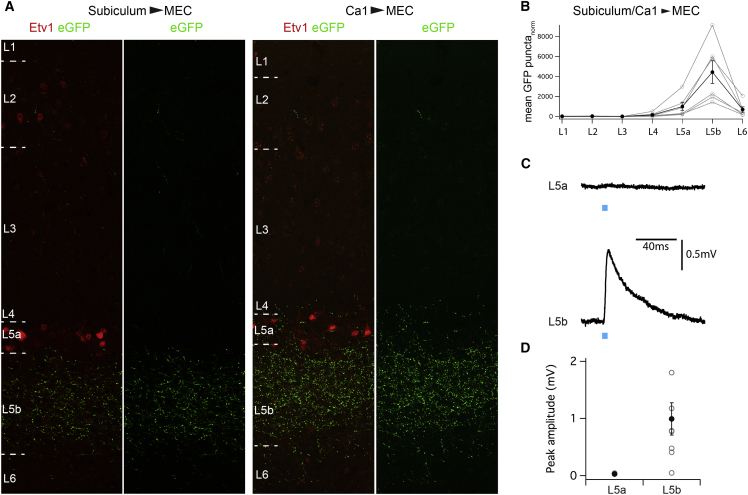
Hippocampal Outputs Preferentially Target Neurons in L5a (A) Putative synaptic terminals labeled in deep layers following expression of synaptophysin-eGFP in the dorsal subiculum (left) or CA1 (right). (B) Density of putative synaptic terminals plotted as a function of layer following injection of synaptophysin-eGFP into the subiculum or CA1. Filled circles indicate the population mean (± SEM). (C) Examples of responses of neurons in L5a and L5b following light stimulation to activate terminals of subicular neurons infected with AAV-ChR2-mCherry. (D) Mean response amplitudes for neurons from L5a and L5b. Filled circles indicate the population mean (± SEM).

To establish whether the specificity in afferent targeting of layer 5b extends to inputs from the hippocampal formation, we investigated the distribution of labeled terminals in the MEC following injection of AAV-synaptophysin-eGFP into the CA1 (n = 3) or subicular (n = 3) regions of the hippocampus (Figures S5A–S5D). The distribution of labeled synaptic terminals in the MEC differed between layers (p = 5.9 × 10^−8^, ANOVA) and was independent of the injection site (p = 0.1, ANOVA). Strikingly, we found that synaptophysin-eGFP labeling was enriched >4-fold in L5b compared to L5a (Figures 6A and 6B) (p = 0.009, paired t test). Preferential targeting of L5b over L5a was similar following injections of AAV-synaptophysin-eGFP (Figure 6A) into the subiculum and CA1 (Figure 6B).

To test whether the preferential targeting of hippocampal projections to L5b results in different functional connectivity, we injected AAV expressing ChR2 into the dorsal subiculum and evaluated responses of neurons in each layer to light stimulation (Figures S5E, S5F, 6C, and 6D). We found that activation of inputs to neurons in L5b generated EPSPs in seven of eight tested neurons (mean amplitude 0.99 ± 0.28 mV) (Figures 6C and 6D), but not in any of 6 projection neurons in L5a (mean amplitude 0.03 ± 0.01 mV, p = 0.012, unpaired t test). Responses of L5b neurons to subicular input were abolished by glutamatergic antagonists and had properties consistent with a monosynaptic connection (Figures S5J–S5J). Together these data indicate that glutamatergic hippocampal projections to the MEC preferentially target neurons in L5b.

## Discussion

**Figure 7:**
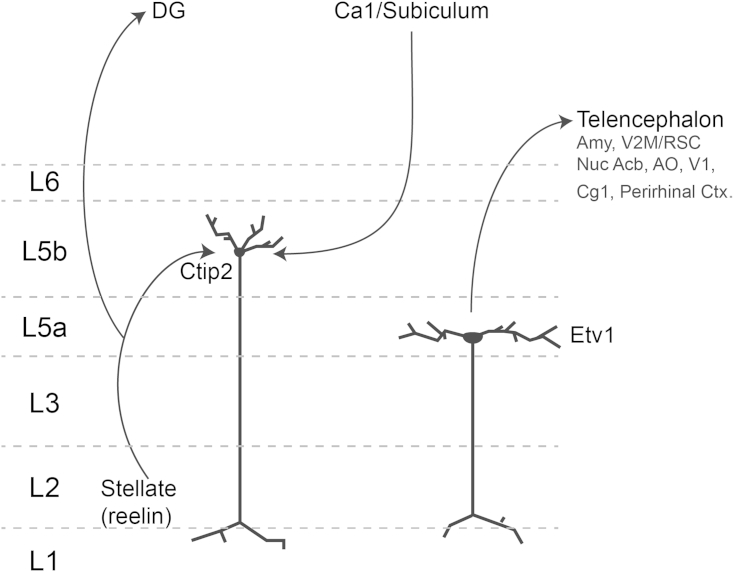
Anatomical Segregation of Input and Output to Deep Layers of the MEC Inputs to deep layers from L2SCs and from the hippocampal formation target neurons in L5b. Outputs from deep layers to telencephalic structures originate in L5a.

Spatial signals in entorhinal-hippocampal circuits contribute to cognitive functions implemented by diverse brain structures. Our results suggest fundamental modifications to the classical view of the deep layers of the MEC as a simple relay of hippocampal outputs to other parts of the brain (Figure 7). First, we find that the distinct molecular identity of neurons in layers 5a and 5b maps onto striking differences in their projections. Thus, for the major telencephalic targets of the MEC, projections originate from neurons in L5a and not L5b. Second, we find that L2SCs, but not L2PCs, have numerous synaptic terminals in the deep layers. These terminals make excitatory connections to neurons in L5b but not to projection cells in L5a. This inter-laminar pathway provides a route for spatially rich signals in L2 to directly influence neurons in deep layers without first passing through the hippocampal circuit. Third, we find that hippocampal input to the MEC preferentially targets neurons in L5b. Our results lead to a new view of the deep layers of the MEC (Figure 7), according to which neurons in L5b integrate inputs from the hippocampus and superficial MEC, while neurons in L5a send outputs to telencephalic structures. Rather than superficial and deep layers acting as independent relays, our results suggest that their interactions may determine computations carried out by the MEC and that these interactions take place between molecularly defined sub-populations of superficial and deep layer neurons.

Consideration of differences between the molecular and synaptic organization of deep layers of the MEC that we establish here, and other cortical regions with different cognitive functions, suggests a unique molecular logic for assembly of deep layers of the MEC. First, IT projection neurons in other cortical regions are found throughout layer 5, whereas in the MEC they are found in L5a, but not L5b. Second, in other cortical regions, Etv1 labeling is found throughout layer 5 but does not segregate with particular projection targets (Yoneshima et al., 2006). In contrast, in MEC Etv1 consistently labels neurons in L5a, which have extensive IT projections, but is not found in layer 5b. Third, in other cortical regions, neurons labeled by Ctip2 and Etv1 are intermingled (Lickiss et al., 2012), whereas in the MEC they are separated. The more discrete sub-layer organization of the MEC may establish the distinct afferent connectivity that we describe for layers 5a and 5b. Thus, the relative lack of overlap between the basal dendrites of neurons in L5a and L5b of the MEC may ensure each neuronal population samples different axonal inputs. In contrast, Ctip2- and Etv1-positive neurons in other cortical regions may be positioned to sample common synaptic inputs. For example, in the neocortex, both IT and other projection neurons receive input from L2/3 (Anderson et al., 2010). Downstream molecular pathways of Ctip2 and Etv1 could be important for the afferent input specificity that we identify here. For example, Ctip2 is an activator of a secreted molecule Sonic hedgehog (Simon et al., 2012), which promotes the formation of L2/3 contacts onto Ctip2 positive L5b neurons in sensorimotor cortex (Harwell et al., 2012). Thus, while sharing similarities to the neocortex, deep layers of the MEC differ in ways that may be critical for the specialized computations carried out by the MEC.

Do spatially rich signals from L2 influence grid firing or extra-hippocampal output from deep layers of the MEC? Previously, observations of cells in deep layers with grid firing fields could only be explained by independent superficial and deep grid generators (Sargolini et al., 2006). This appears at odds with the higher density of grid cells in the superficial compared to deep layers but nevertheless would be compatible with models in which superficial layer grid cells inherit their spatial firing from deeper layers (Tocker et al., 2015). By demonstrating that information encoded by L2SCs, but not L2PCs, can directly influence principal cells in deep layers of the MEC, our results suggest a circuit mechanism for grid activity patterns in deep layers to be inherited from the more numerous grid cells in L2, or for grid fields to be generated through feedback loops operating across all layers. Neurons receiving input from L2SCs do not appear able to directly influence IT structures but may influence thalamic nuclei. This is consistent with reports of projections from MEC to the thalamus in monkeys (Saunders et al., 2005). Neurons in L5b may also have local actions within the MEC, either onto output neurons in L5a or onto hippocampally projecting neurons in layers 2 and 3. Consistent with this possibility, L5b neurons have axon collaterals that project into more superficial layers (c.f. Figure 4) (Canto et al., 2008). For each of these scenarios, our data establish L2SCs, rather than L2PCs, as critical for feedback from superficial to deep layers.

Deep layers of the MEC are conventionally considered as a relay of hippocampal signals to the neocortex. A straightforward interpretation of previous data is that the same neurons receive input from the hippocampus, integrate this input, and generate an output appropriate for downstream structures. Our results introduce two substantial modifications to this view. First, the input and output components of deep layers are segregated. Neurons in layer 5b preferentially receive inputs from the hippocampus and superficial MEC, while neurons in layer 5a appear to exclusively mediate outputs to telencephalic structures. Second, hippocampal signals are integrated with output from superficial layers of the MEC. This may allow integration of hippocampal location estimates with path integrator outputs from grid cells. Testing models for computation in deep layers will require establishing routes for interaction between Ctip2 L5b neurons and Etv1 L5a neurons and rules for plasticity and integration by each circuit component.

In conclusion, our results demonstrate precise connectivity of molecularly defined neuron types in the MEC. Molecular markers of neuronal populations with distinct efferent and afferent connectivity will enable future dissection of their roles in spatial computation and long-term memory. Thus, it should in the future be possible to address the respective roles of L5a and L5b in navigation and spatial memory and to establish the respective roles of inputs from the hippocampus and from superficial layers of the MEC.

## Experimental Procedures

### Mouse Strains

All animal experiments were approved by the University of Edinburgh animal welfare committee and were performed under a UK Home Office project license. For full details of mouse strains and their maintenance, see the Supplemental Experimental Procedures. Briefly, *Sim1*:*Cre* mice were generated by GenSat and obtained from MMRRC (strain name: Tg(Sim1cre)KJ21Gsat/Mmucd). *Wfs1*:*CreER* (Wfs1-Tg3-CreERT2) mice were generated by the Allen Institute for Brain Sciences and obtained from Jackson Labs (Strain name: B6;C3-Tg(Wfs1-cre/ERT2)3Aibs/J; stock number:009103). RCE:loxP (R26R CAG-boosted EGFP) mice were generated as described in Miyoshi et al. (2010). C57Bl6J mice were obtained from the Jackson Laboratories and used in retrograde mapping experiments and as breeders to maintain the transgenic lines heterozygous for the transgene insertion locus. 6- to 10-week-old male and female mice were used in all experiments.

### Tissue Processing and Immunohistochemistry

For immunohistochemistry, anesthetized mice were perfused with cold PBS followed by 4% cold paraformaldehyde (PFA) or formalin. After overnight fixation in cold PFA, brains were washed with PBS and transferred to 30% sucrose solution prepared in 0.1 M PB for 48 hr. 50- to 60-μm-thick horizontal or sagittal brain slices were cut using a freezing microtome. Prior to the application of primary antibodies, slices were blocked in 2% BSA or 5% Normal Goat Serum (NGS) in 0.3% PBS-T (Triton) for 2 to 3 hr at 4°C. Slices were transferred to primary antibody solution prepared in 0.2% BSA or 5% NGS in 0.3% PBS-T for 16 to 20 hr. Next, slices were washed in 0.3% PBS-T 4 times for 20 min and transferred to secondary antibody solution. After overnight incubation and four times washes in 0.3% PBS-T, slices were mounted on glass slides and coverslipped using Mowiol. The following primary antibodies were used: rabbit anti-Etv1 (gift from Thomas Jessell (Arber et al., 2000), rat anti-Ctip2 (Abcam, ab18465, 1:1,000), mouse anti-reelin (Millipore MAB5364, 1:1,000), mouse anti-reelin (MBL D223-3, 1:200), rabbit anti-GFP (Invitrogen, 1:500), chicken anti GFP (Abcam ab13970, 1:5,000), mouse anti-parvalbumin (SWANT PV235, 1:3,000), and rabbit anti-calbindin D-28k (SWANT CB-38, 1:2,500). NeuroTrace 640/660 (Invitrogen, 1:800) and all secondary antibodies were obtained from Invitrogen. A heat-mediated antigen retrieval procedure was applied on tissue stained with Ctip2 antibody.

For reconstruction of the morphology of neurons following patch-clamp recordings, cells were filled with biocytin and following recording slices were fixed overnight at 4°C in 4% PFA or formalin. The next day, slices were washed with PBS three times and transferred to streptavidin-Alexa488 (Invitrogen S-11223, 1:1,000) or streptavidin-Texas Red (Invitrogen S-872, 1:1,000) and NeuroTrace solution prepared in 0.3% PBS-T for 16 to 20 hr. Slices were washed in PBS three times and mounted on glass slides with Mowiol or Vectashield.

### Injection of Dyes and Viruses

Mice were anesthetized with isoflurane and mounted in a stereotaxic frame, and a small craniotomy was made above the target region. For viral transduction of entorhinal neurons, ∼200 nl of one of the following viruses was injected through a glass pipette: AAV-FLEX-rev-ChR2mCherry, which expresses ChR2-mCherry from a CAG promoter (Atasoy et al., 2008, AddGene 18916) (titer: 2.2 × 10^14^ cp/ml, measured by qPCR. cp stands for capsid particle); AAV-FLEX-GFP, which expresses GFP from a CBA promoter (Murray et al., 2011) (titer: 1.5 × 10^12^ cp/ml); or AAV-FLEX-synaptophysin-eGFP, which expresses synaptophysin-eGFP from a CBA promoter (modified from Groh et al., 2008) (titer: 1.2 × 10^12^ cp/ml). To make AAV-FLEX-synaptophysin-eGFP, the synaptophysin-eGFP sequence (Groh et al., 2008) was excised from pAM synaptophysin EGFP and cloned into the NotI and EcoRV sites of pAM FLEX (Murray et al., 2011). For all FLEX viruses, Cre-dependent inversion of the coding region is required for expression to occur (Atasoy et al., 2008). Plasmids were packaged into AAVs with a chimeric 1/2 serotype as described previously (McClure et al., 2011).

Strategies for targeting of viral injections to the MEC are described in the Supplemental Experimental Procedures. Possible spread of expression beyond layer 2 of MEC was examined in slices used for electrophysiological assessment of connectivity and synaptic terminal labeling experiments. Data from mice where virus infectivity was observed in layer 5a or parasubiculum were discarded.

For retrograde labeling of MEC projection neurons, cholera toxin beta subunit conjugates CTB-Alexa488 or CTB-Alexa555 (Invitrogen, 0.1%) or Fast Blue (Polysciences 17740-1) were injected in the reported coordinates (see Figure S1). Animals were used in subsequent experiments 1 to 2 weeks after recovery. The location of the fluorescent signals at the injection sites and needle tracts were imaged after staining tissue with NeuroTrace. Using these images, injection sites were then mapped through comparisons with the reference sections from the Mouse Brain Atlas (Paxinos and Franklin, 2008).

For injections into the dorsal subiculum and CA1 regions of the hippocampus, we made craniotomies directly above the targeted site. Injection coordinates were calculated relative to bregma (subiculum, X: +1.3 mm, Y: −3.0 mm, Z: −1.5 mm; Ca1, X: +1.3 mm, Y: −1.5 mm, Z: −1.3). We injected 200 nl of either AAV2/1-CBA-synaptophysin-eGFP (Groh et al., 2008), AAV-hSyn-hChR2(H134R)-mCherry (UNC vector core, Karl Deisseroth virus stock), or AAV-CAG-ChR2-Venus (Vector Biolabs, AddGene: 20071). In the same surgery, L5a neurons projecting to V2M/RSC were retrogradely labeled with cholera toxin beta subunit conjugates as described above.

### Image Acquisition and Data Analysis

All images were acquired using a Nikon A1 confocal microscope and NIS elements software. For co-localization studies to assess molecular identities of *Sim1*:*Cre* and *Wfs1*:*CreER* cells, immunostained tissue was imaged using a 20× air objective with a pinhole diameter set to 1 Airy unit and using the z stack function to acquire an image file that encompasses 15–20 μm tissue depth. The co-localization measurements of fluorescent markers were then carried out manually. Cell body size measurements based on diameter calculations for Figure 1 was made using the NIS elements software (Nikon).

### Methods for Quantification of Fractions of Labeled Cells

Regions of interest (ROIs) for quantification of marker expression in *Sim1*:*Cre* and *Wfs1*:*CreER* mice in Figure 2 were selected where the expression of the reporter virus/gene was highest and mostly confined to the dorsal half of the MEC. All neurons within the plane of view were counted and analyzed for colocalization. NeuroTrace and Etv1 or Ctip2 colocalization experiments reported in Figure 1 were also performed manually. The ROI for Etv1 quantifications were localized in mid to lateral MEC sections where L5a is widest. When the layer distribution of backlabeled neurons were quantified in Figure S1, ROIs were selected where the density of backlabeled neurons were the highest and in regions where labeled cells were detected in more than one layer.

For quantification of putative synaptic terminals, synaptophysin-eGFP viral construct injected brain slices were imaged using a 40× oil objective with an additional 2.0× digital zoom. A single focal plane was imaged from multiple tissue sections from each injected brain. The number of sections imaged and analyzed per brain depended on the dorsoventral (for horizontal sections) and mediolateral (for sagittal sections) coverage of the virus infection, which usually spanned two to four slices, 120 μm apart from each other. Measurements for *Sim1*:*Cre* mice were obtained by analyzing two to four horizontal tissue sections from each mouse. Measurements for *Wfs1*:*CreER* mice were obtained by analyzing three tissue sections from each mouse. Measurements for projections from CA1 and the dorsal subiculum were obtained from three or four sagittal sections per mouse. The analysis of synaptophysin-eGFP puncta counts was made using Imaris software (Bitplane, Oxford Instruments). For analysis of projections from L2, the analysis was limited to a 250- to 400-μm-wide (in the mediolateral plane) segment of the brain section where synaptophysin-eGFP positive cell density in L2 was the highest. Within this segment, a region of interest in each layer was created using the Surface function in Imaris and with the Neurotrace and Etv1 stainings as a guide for delineating layer borders. GFP puncta were counted in these ROIs and normalized to the ROI area to calculate puncta density. The Imaris Spots function with a size and intensity filter was used to assign puncta as spots. Manual post-checks were performed to eliminate potential false positives (debris, labeling of axon segments) and negatives. Puncta densities for L3, L4, L5a, L5b, and L6 were then normalized to the number of infected neurons in the ROI in layer 2. Data from all sections from each mouse were averaged to generate mean values used for subsequent statistical comparisons. Analysis of projections from CA1 and the subiculum was carried out in a similar way except that AAV-CBA-synaptophysin-eGFP was used and normalization to the number of infected neurons was not carried out.

For the reconstruction of biocytin-filled neurons, confocal images of filled neurons were acquired using a 20× air objective using the z stack function to acquire an image file that encompasses the entire depth of the cell body and processes. When the size of the neuron exceeded the image field, multiple images were taken and then stitched after reconstructions. Reconstructions of filled neurons and cell body surface area measurements of recorded neurons were carried out using Imaris (BitPlane) and Neurolucida (MBF Bioscience).

### Electrophysiological Recordings

Preparation of brain slices and electrophysiological recordings were carried out as described previously (Garden et al., 2008, Pastoll et al., 2012b, Pastoll et al., 2013) and in full in the Supplemental Experimental Procedures. Briefly, sagittal and horizontal brain slices were prepared from 8- to 10-week-old male and female mice. Whole-cell patch-clamp recordings were made from neurons in all layers and the full mediolateral extent of the MEC. Recordings were mainly limited to the dorsal half of the MEC (Figure S4). Experiments to test hippocampal inputs to the MEC cells were conducted while inhibition was blocked by addition of picrotoxin (final concentration 50 μM) to the extracellular recording solution. Recorded cells were identified using criteria described previously (Gonzalez-Sulser et al., 2014, Pastoll et al., 2012a).

Fluorescently labeled cells in *Sim1*:*Cre* and *Wfs1*:*CreER* mice were identified for recording by their expression of mCherry or GFP. For electrophysiology experiments, *Sim1*:*Cre* mice were injected with AAV-FLEX-rev-ChR2mCherry or AAV-FLEX-GFP. Wfs1CreER mice were injected with AAV-FLEX-GFP. Light of wavelength 470 nm from an LED (Thor Labs) attached to the epiflourescence port of the microscope was used to activate L2SCs expressing ChR2-mCherry. Light pulses of duration 3 ms and at a range of intensities (0.48, 0.86, 1.21, 1.56, 1.88, 2.22, 4.61, 7, 9.24, 11.4 mW) were applied after stable recordings were established. Stimuli were repeated five times. When stated, the following pharmacological agents were bath applied in the standard extracellular solution (final concentrations in μM): NBQX 5, APV 50, and picrotoxin 50 (all from Abcam).

### Data Analysis and Statistics

Data are presented as mean ± SEM. Data analysis and statistics used R (http://www.r-project.org), Excel (Microsoft), and built-in and custom routines in IGORpro (Wavemetrics). Comparisons between groups used ANOVA or Student’s t test unless indicated otherwise. Post hoc analysis of synaptophysin-eGFP labeling in *Sim1*:*Cre* and *Wfs1*:*Cre* mice involved only a single planned comparison between densities in L5a and L5b, and therefore, unadjusted p values from paired Student’s t tests are reported. Post hoc comparison of differences in overall labeling between *Sim1*:*Cre* and *Wfs1*:*Cre* mice was not planned for any particular layer, and therefore the reported p value reported for L5b is adjusted using the method of Benjamini and Hochberg (1995). Evaluation of differences in proportions used a Z test.

Spike latencies for ChR2-mCherry expressing neurons and PSP latencies for putative postsynaptic neurons were measured, respectively, from the onset of the light stimulus until the membrane potential crossed a threshold of −40 mV and to the time point where 20% of the peak PSP amplitude was reached. Means and SDs were calculated for each cell from responses to four to five stimuli. The mean peak PSP amplitude was calculated as the average from four to five responses of the difference between a baseline period before light stimulation and a 1 ms window centered on the peak of the mean of all of the EPSPs. Unless indicated otherwise, synaptic potentials shown in figures are averages of four to five responses.

## Author Contributions

Conceptualization, M.F.N. and G.S.; Methodology, M.F.N. and G.S.; Formal Analysis, G.S., D.L.F.G., D.C.M., and M.F.N.; Investigation: G.S, H.P., D.L.F.G., and C.M.; Writing – Original Draft, M.F.N. and G.S.; Writing – Review and Editing, M.F.N. and G.S.; Supervision, M.F.N. and G.S.; Funding Acquisition: M.F.N. and G.S.; Project Administration: M.F.N.

## Supplemental Information

Document S1. Figures S1–S5 and Supplemental Experimental Procedures

Document S2. Article plus Supplemental Information
