# Molecularly Defined Circuitry Reveals Input-Output Segregation in Deep Layers of the Medial Entorhinal Cortex

**DOI:** 10.1016/j.neuron.2016.11.011

**Published:** 2016-11-23

**Authors:** Gülşen Sürmeli, Daniel Cosmin Marcu, Christina McClure, Derek L.F. Garden, Hugh Pastoll, Matthew F. Nolan

(Neuron *88*, 1040–1053; December 2, 2015)

In the original article, the title of Figure 6 was written as “Hippocampal Outputs Preferentially Target Neurons in L5a.” The correct title should be “Hippocampal Outputs Preferentially Target Neurons in L5b.” This has been corrected online. The authors regret the error.

