# Supplementary material for: Molecularly Defined Circuitry Reveals Input-Output Segregation in Deep Layers of the Medial Entorhinal Cortex
Source: Neuron. 2015 Dec 2;88(5):1040–53. doi: 10.1016/j.neuron.2015.10.041 (PMC4675718; doi:10.1016/j.neuron.2015.10.041)
Supplement: Document S1. Figures S1–S5 and Supplemental Experimental Procedures [file mmc1.pdf]

Neuron, Volume *88*

Supplemental Information

Molecularly Defined Circuitry Reveals Input-Output Segregation in Deep Layers of the Medial Entorhinal Cortex

Gülşen Sürmeli, Daniel Cosmin Marcu, Christina McClure, Derek L.F. Garden, Hugh Pastoll, and Matthew F. Nolan

Molecularly defined circuitry reveals input-output segregation in deep layers of the medial entorhinal cortex

Gülşen Sürmeli[1], Daniel Cosmin Marcu[1], Christina McClure[1], Derek L.F. Garden[1], Hugh Pastoll[1], Matthew F. Nolan[1]*

[1]Centre for Integrative Physiology, University of Edinburgh, Hugh Robson Building, Edinburgh, EH8 9XD, United Kingdom

*Correspondence: mattnolan@ed.ac.uk (M.F.N.)

Supplemental Experimental Procedures

Mouse strains. All animal experiments were approved by the University of Edinburgh animal welfare committee, and were performed under a UK Home Office project license. *Sim1:Cre* mice were generated by GenSat and obtained from MMRRC (strain name: Tg(Sim1cre)KJ21Gsat/Mmucd). *Wfs1:CreER* (Wfs1-Tg3-CreERT2) mice were generated by the Allen Institute for Brain Sciences and obtained from Jackson Labs (Strain name: B6;C3-Tg(Wfs1-cre/ERT2)3Aibs/J; stock number:009103). *RCE:loxP* (R26R CAG-boosted EGFP) mice were generated as described in (Miyoshi et al., 2010). C57Bl6J mice were obtained from the Jackson Laboratories and used in retrograde mapping experiments and as breeders to maintain the transgenic lines heterozygous for the transgene insertion locus. 6-10 week old male and female mice were used in all experiments. For a subset of experiments where the *Wfs1:CreER* line was characterized, *Wfs1CreER* ; *RCE:loxP* crosses were used. These mice received tamoxifen treatment to boost Cre activity. Tamoxifen (Sigma, 20mg/ml in corn oil) was administered on 3 consecutive days by intraperitoneal injections (daily dose: 75mg tamoxifen/kg bodyweight) one week before the experiments. No harmful effects of the viral expression or tamoxifen treatment was observed. Some global glial expression of eGFP was observed upon tamoxifen injection in *Wfs1:CreER* ; *RCE:loxP* reporter crosses. When Sim1:Cre was crossed to the *RCE:loxP* line, GFP expression was observed in L5a and in non-stellate cells in L2 of the MEC. This is in contrast to the stellate cell specific expression in adult mice after viral injections of the Cre dependent reporter construct and suggests Cre activity in non-stellate L2 neurons in earlier

developmental (embryonic or early postnatal) stages. SC-specific expression of GFP and ChR2-mCherry was achieved by using 6-10 week old mice and Cre dependent viruses with optimized titres and injection methods (see section: Injection of dyes and viruses).

Targeting of injections to the MEC. To target the MEC, a craniotomy was made 3.5 to 3.65 mm lateral to bregma between the transverse sinus and lambdoid suture (4.2-4.4 mm caudal to bregma). The injection pipette was at an 8-9 degree rostral angle to the dorsoventral plane (See Figure S2) and was slowly lowered until a slight bending indicated the approach to the caudal surface of the brain. This point usually corresponded to a 2.0-2.6 mm depth from the surface of the brain. At this point the needle was retracted by about 200 μm to a position in layer 2 of the MEC. 3 mins after the release of the virus the needle was slowly retracted. Animals were used in experiments 2-3 weeks after recovery. With this strategy we did not detect labeling of neurons in parasubiculum or deep layers of the MEC (Figure 2 and Figure S2C).

Electrophysiological recordings. Sagittal and horizontal brain slices were prepared from 8-10 week old male and female mice as described previously (Garden et al., 2008; Pastoll et al., 2013; Pastoll et al., 2012b). Whole-cell patch-clamp recordings were made from neurons in all layers and the full mediolateral extent of the MEC. Recordings were mainly limited to the dorsal half of the MEC (Figure S4). For preparation of slices the cutting solution had composition (mM): NaCl 86, NaH_2PO_4 1.2, KCl 2.5, $NaHCO_3$ 25, Glucose 25, Sucrose 50, $CaCl_2$ 0.5, $MgCl_2$ 7. After the slicing and throughout the experiment slices were maintained in a solution with the following components (mM): NaCl 124, NaH_2PO_4 1.2, KCl 2.5, $NaHCO_3$ 25, Glucose 25, $CaCl_2$ 2, $MgCl_2$ 1. Experiments to test hippocampal inputs to the MEC cells were conducted while inhibition was blocked by addition of picrotoxin (final concentration 50 μM) to the recording solution. For patch-clamp recordings the intracellular solution had composition (mM): K Gluconate 130; KCl 10, HEPES 10, $MgCl_2$ 2, EGTA 0.1, Na_2ATP 2, Na_2GTP 0.3 NaPhosphocreatine 10, biocytin 5.4. An experimentally measured liquid junction potential of 12.9 mV was not corrected for. Current-clamp experiments were

carried out with series resistance ≤ 30 MΩ and with bridge-balance and pipette capacitance neutralization applied.

Recorded cells were identified using criteria described previously (Gonzalez-Sulser et al., 2014; Pastoll et al., 2012a). Briefly, L2SCs were distinguished from pyramidal cells by their characteristic large sag response to current steps and by their distinct clustered patterns of action potentials upon current injection. We excluded from analysis recordings from putative interneurons, which could be identified as fast spiking (FS) or low threshold spiking (LTS) (Gonzalez-Sulser et al., 2014). Identification based on the morphology of reconstructed neurons was consistent with our electrophysiological criteria.

Fluorescently labeled cells in *Sim1:Cre* and *Wfs1:CreER* mice were identified for recording by their expression of mCherry or GFP. For electrophysiology experiments *Sim1:Cre* mice were injected with AAV-FLEX-rev-ChR2mCherry or AAV-FLEX-GFP. *Wfs1:CreER* mice were injected with AAV-FLEX-GFP. Light of wavelength 470 nm from an LED (Thor Labs) attached to the epiflourescence port of the microscope was used to activate L2SCs expressing ChR2-mCherry. Light pulses of duration 3 ms and at a range of intensities (0.48, 0.86, 1.21, 1.56, 1.88, 2.22, 4.61, 7, 9.24, 11.4 mW) were applied after stable recordings were established. Stimuli were repeated 5 times.

When stated the following pharmacological agents were bath applied in the standard extracellular solution (final concentrations in µM): NBQX 5, APV 50, picrotoxin 50 (all from Abcam). Recordings with 3 ms single pulse light stimulation every 30 seconds were acquired for 5 mins before the application of drugs to establish a stable baseline. Drugs were applied for a minimum of 10 mins and drug effects were monitored through 3 ms single pulse light stimulation every 30 seconds. After the drug application, a series of current steps were applied to confirm the stability of the recording.

A

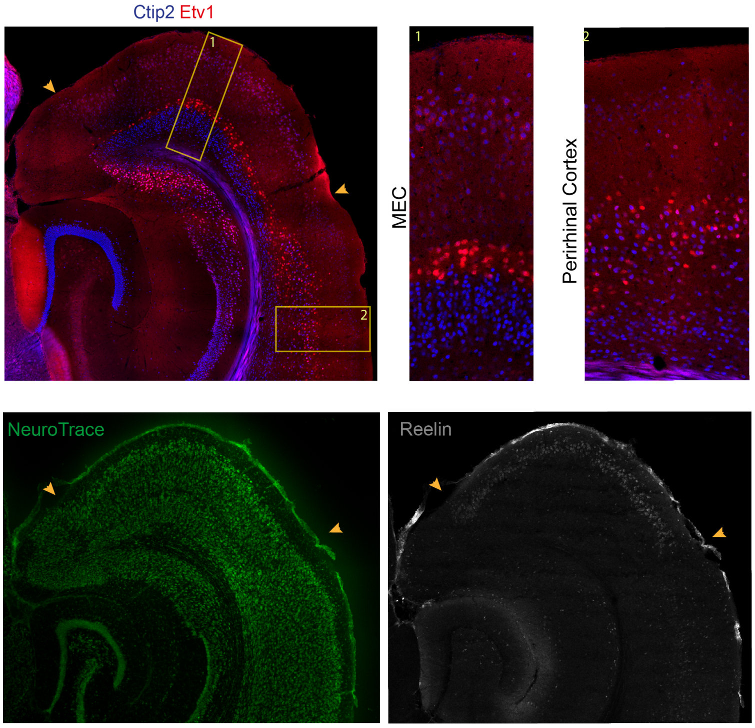

B

Target brain regions	Distribution of back-filled neurons in MEC (% ± s.e.m.)			Injection coordinates	Number of mice and cells
	L5a	L5b	L6		
Perirhinal Ctx	97 ± 1.3	0.6 ± 0.5	2.4 ± 1.3	X: 4.6; Y: -1.73; Z: 2.3 - 2.1	n = 3 mice 171 cells
NucAcb/AO	99.8 ± 0.2	0.2 ± 0.2	0	X: 0.7; Y: +1.15; Z: 4.7 - 4.5	n = 5 mice, 662 cells
RSC/V2M	98.4 ± 0.6	0.2 ± 0.2	1.4 ± 0.5	X: 1.0; Y:-3.5; Z: 0.8 - 0.5	n = 4 mice, 600 cells
Cg1	97.7 ± 1.9	0.6 ± 0.5	1.7 ± 1.4	X: 0.45; Y: +1.0; Z: 1,3 - 1.0	n = 3 mice, 230 cells
V1	98.0 ± 0.9	0.4 ± 0.3	1.5 ± 0.6	X: 2.7; Y: -3.3; Z: 0.7	n = 3 mice, 342 cells
Hippocampus	25.2 ± 7.3	1.9 ± 0.2	72.9 ± 7.3	X: 2.5; Y: -2.3; Z: 1.7 and X: 1.48; Y: -2.3; Z: 2.3 - 1.8	n = 4 mice, 617 cells
Amygdala	88.5 ± 2.6	2.1 ± 0.5	9.4 ± 2.4	X: 2.85; Y: -1.0; Z: 5.5 -5.0	n = 6 mice, 1117 cells
Thalamic nuclei	0	61.8 ± 4.5	38.2 ± 4.5	X:0.7 - 1.0; Y: -0.8; Z: 2.5 - 3.5 and X: 0.8; Y: -1.2; Z: 2.4	n = 2 mice, 195 cells
M1	0	0	0	X: 1.8; Y: +1.4; Z: 0.7	n = 2

C

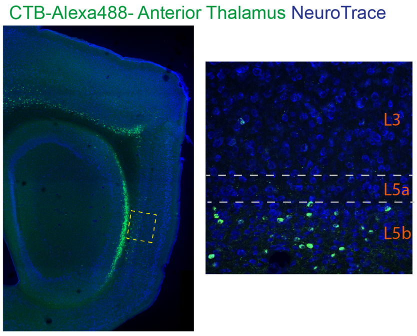

Supplemental Figure 1 (refers to Figure 1).
(A) Horizontal brain sections immunostained for Ctip2 (blue) and Etv1 (red) (upper left). The boxed areas are shown at higher magnification (upper right) to illustrate the spatial segregation of Ctip2+ and Etv1+ cells in the MEC (1) and the intermingling of populations labeled by these markers in the nearby perirhinal cortex (2). Arrowheads show the borders of the entorhinal cortex. These borders are determined by the neuronal (NeuroTrace, green) and reelin (white) immunostainings of adjacent sections (lower panels).
(B) Table summarizing the layer identity of projection neurons that are labeled following retrograde dye injections targeted to the listed areas. Note that back-labeled L5b neurons were not encountered except for a few cells found in medial sections of MEC after thalamic injections. Abbreviations: Perirhinal Ctx: Perirhinal Cortex; NucAcb: Nucleus Accumbens; AO: Anterior Olfactory Area; RSC: Retrosplenial Cortex, V2M: Secondary visual cortex medial area; Cg1: Cingulate Cortex 1; V1: Primary visual cortex, M1: Primary motor cortex; X: lateral distance from midline suture; Y: rostro-caudal distance from Bregma; Z: distance from the surface of the brain. All measurements are in mm.
(C) Example of a sagittal brain section following injection of CTB-Alexa 488 into the anterior thalamus. Inset (boxed area) shows sparsely labeled cell bodies in L5b.

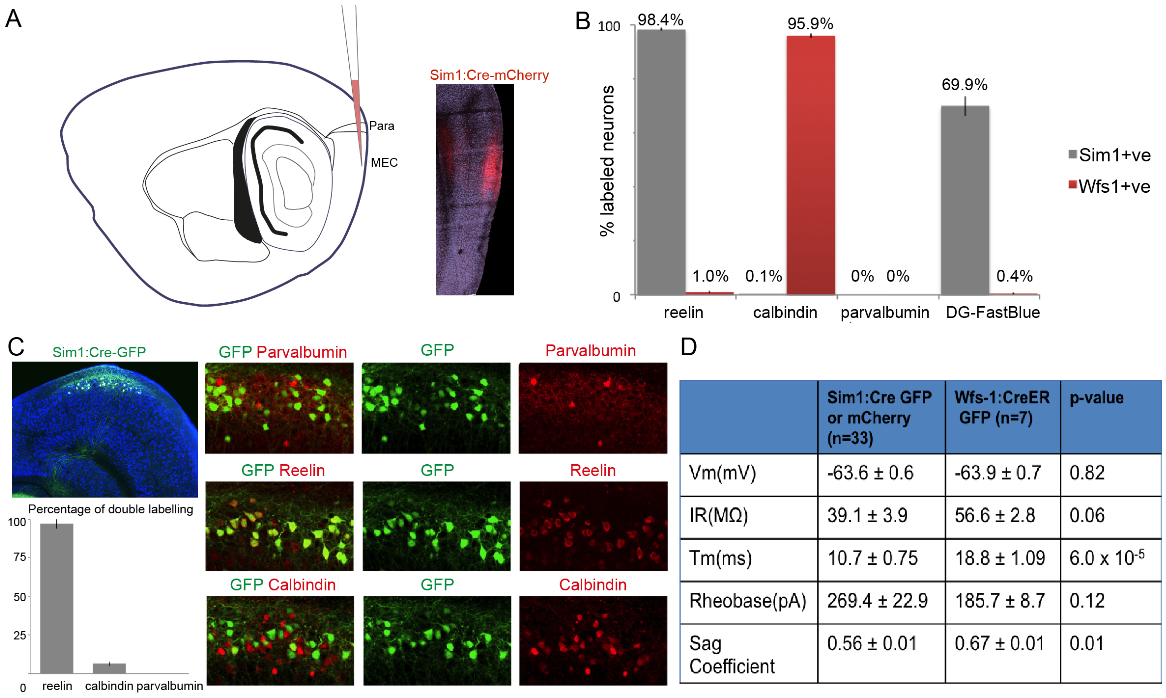

D

	Sim1:Cre GFP or mCherry (n=33)	Wfs-1:CreER GFP (n=7)	p-value
Vm(mV)	-63.6 ± 0.6	-63.9 ± 0.7	0.82
IR(MΩ)	39.1 ± 3.9	56.6 ± 2.8	0.06
Tm(ms)	10.7 ± 0.75	18.8 ± 1.09	6.0×10^{-5}
Rheobase(pA)	269.4 ± 22.9	185.7 ± 8.7	0.12
Sag Coefficient	0.56 ± 0.01	0.67 ± 0.01	0.01

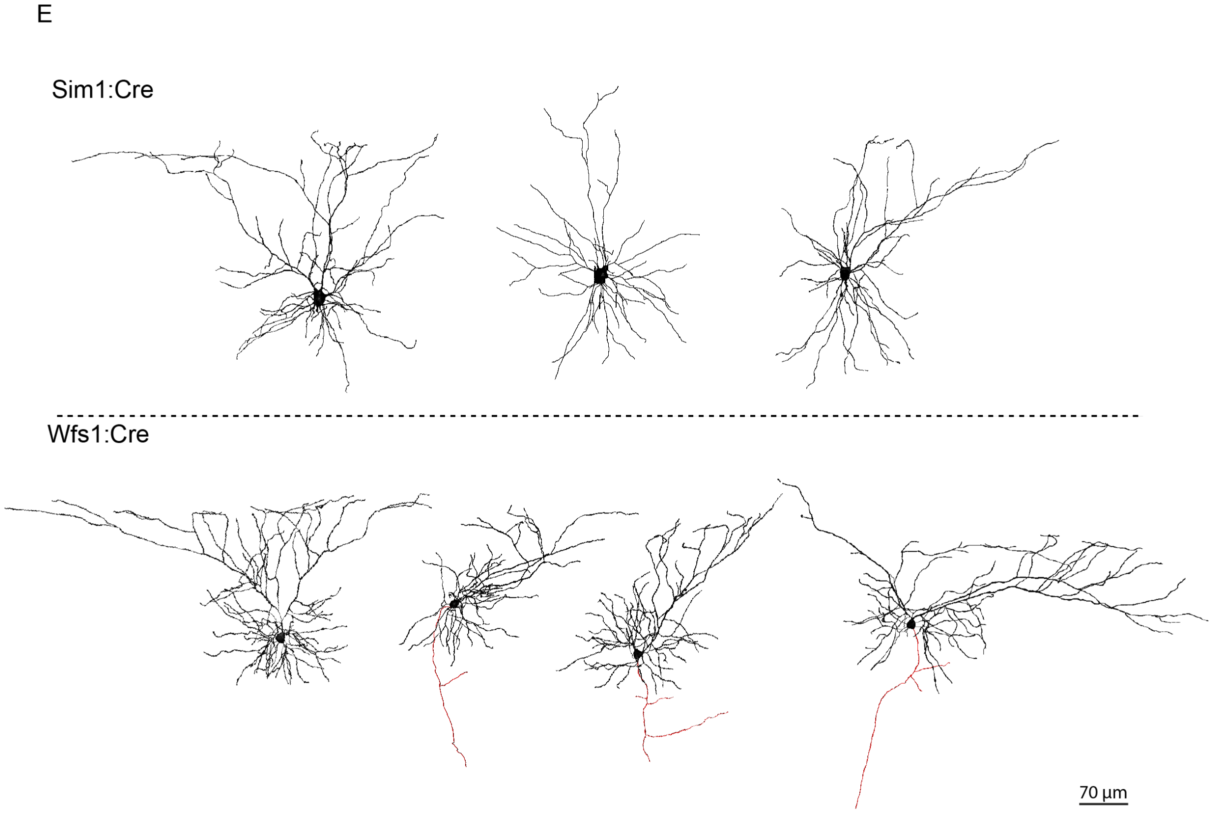

Supplemental Figure 2 (refers to Figure 2).
(A) Injection strategy targeting layers 1 and 2 of the MEC enables specific labeling of layer 2 neurons. Inset shows an example of mCherry fluorescence following injection of AAV-FLEX-rev-ChR2-mCherry into the MEC of a *Sim1:Cre* mouse (Sim1:Cre-mCherry).
(B) Bar charts showing the percentages of Sim1:Cre-mCherry (grey) and Wfs1:CreER-GFP (red) neurons labeled with antibodies against reelin, calbindin and parvalbumin (left three pairs of columns). The right most columns show percentage of Sim1:Cre-GFP (grey) and Wfs1:CreER-GFP (red) neurons co-labeled with the retrograde dye Fast Blue injected into the dentate gyrus (DG-Fast Blue).
Reporter gene expression (mCherry) in L2 of the MEC of *Sim1:Cre* mice following injections of AAV-FLEX-rev-ChR2-mCherry labels cells that are also retrogradely labeled from the DG (69.9 ± 3.8 %, 184 / 285 cells, n = 3 mice) and by immunostaining for reelin (98.4 ± 0.6 %, 1059 / 1072 cells, n = 6 mice) but not calbindin (0.1 ± 0.01%, 23 / 1609 cells, n = 8 mice). Reporter gene expression (GFP) in *Wfs1:CreER* ; *RCE:LoxP* mice labels cells in MEC L2 that are not retrogradely labeled from the dentate gyrus (0.4 ± 0.4 %, 2/399 cells, n=3 mice) or immuno-labeled for reelin (1.0 ± 0.4 %, 11/1085 cells, n = 3, mice) but are positive for calbindin (95.9 ± 0.8 %, 669/697 cells, n = 4 *Wfs1:CreER* ; *RCE:loxP* mice; n=3 virus injected mice).
Note that there is no overlap between reporter gene expression and parvalbumin expression (0%, 0/609 cells, n=5 mice) *in Sim1:Cre* and *Wfs1:CreER* lines (0%, 0/491 cells, n=3 mice). In addition, only a subset of calbindin positive neurons expressed the reporter gene (67.6 ± 5.1 %, 697/1180, n = 4 *Wfs1:CreER* ; *RCE:loxP* mice; n=3 AAV-FLEX-GFP injected *Wfs1:CreER* mice).
(C) Examples of GFP fluorescence, following injection of AAV-FLEX-GFP in the MEC of *Sim1:Cre* mice, and co-labeling with antibodies against parvalbumin, reelin and calbindin. Quantification of co-labeling is shown in the bar chart (reelin/GFP: 97.0 ± 2.1%, n=2 mice, 288/299 cells; calbindin/GFP: 6.4 ± 0.8%, n=3 mice; 73/1418 cells; parvalbumin/GFP: 0% 0/1250 cells, n=2 mice).
(D) Electrophysiological properties of fluorescently labeled neurons in *Sim1:Cre* and *Wfs1:CreER* mice determined from subthreshold membrane potential responses to current injections during whole-cell recordings. Membrane time constant (Tm) and Sag coefficient are significantly different between *Sim:Cre* and *Wfs1:CreER* labeled populations, whereas membrane potential (Vm) and input resistance (IR) are not.
(E) Example reconstructions of fluorescently labeled neurons in *Sim1:Cre* (upper) and *Wfs1:CreER* mice (lower) after filling with biocytin during patch-clamp recordings. Neurons were identified by their expression of the Cre-dependent fluorescent reporter prior to recording. Dendrites and cell bodies are colored in black, axons in red.

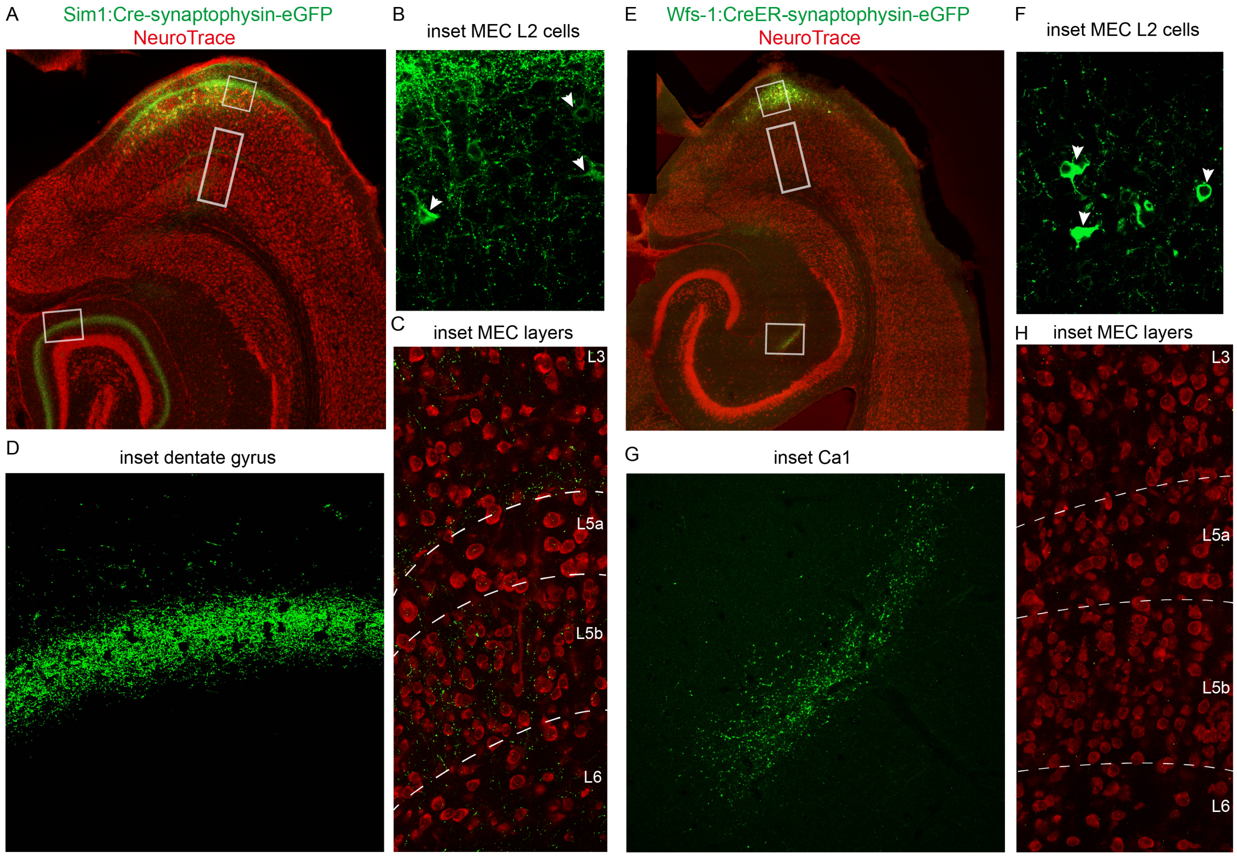

Supplemental Figure 3 (refers to Figure 3).

(A) Example of a horizontal brain sections from a *Sim1:Cre* mouse injected with AAV-FLEX-synaptophysin-eGFP.

(B-D) Higher magnification images of the areas highlighted by boxes in (A). In (B) labeled synaptophysin-EGFP positive cell bodies can be identified (arrowheads). Note also the dense terminal labeling at the border of layers 1 and 2. In (B) and (D) only the eGFP signal is shown. In (C) the dashed lines indicate the layer borders, which were determined based on the size and arrangement of NeuroTrace labeled neurons.

(E) Example of a horizontal section from a *Wfs1:CreER* mouse injected with AAV2/1-CBA-fl-synaptophysin-eGFP.

(F-H) Areas indicated by the boxes in (E) at a higher magnification. In (F) the labeled *Wfs1:CreER* positive cell bodies are indicated by arrowheads. In (F) and (G) only the eGFP signal is shown. Note in (G) that terminal labelling is restricted to the border between stratum radiatum and stratum lacunosum moleculare. In (H) dashed lines indicate layer borders, which were determined based on the size and arrangement of NeuroTrace labeled neurons.

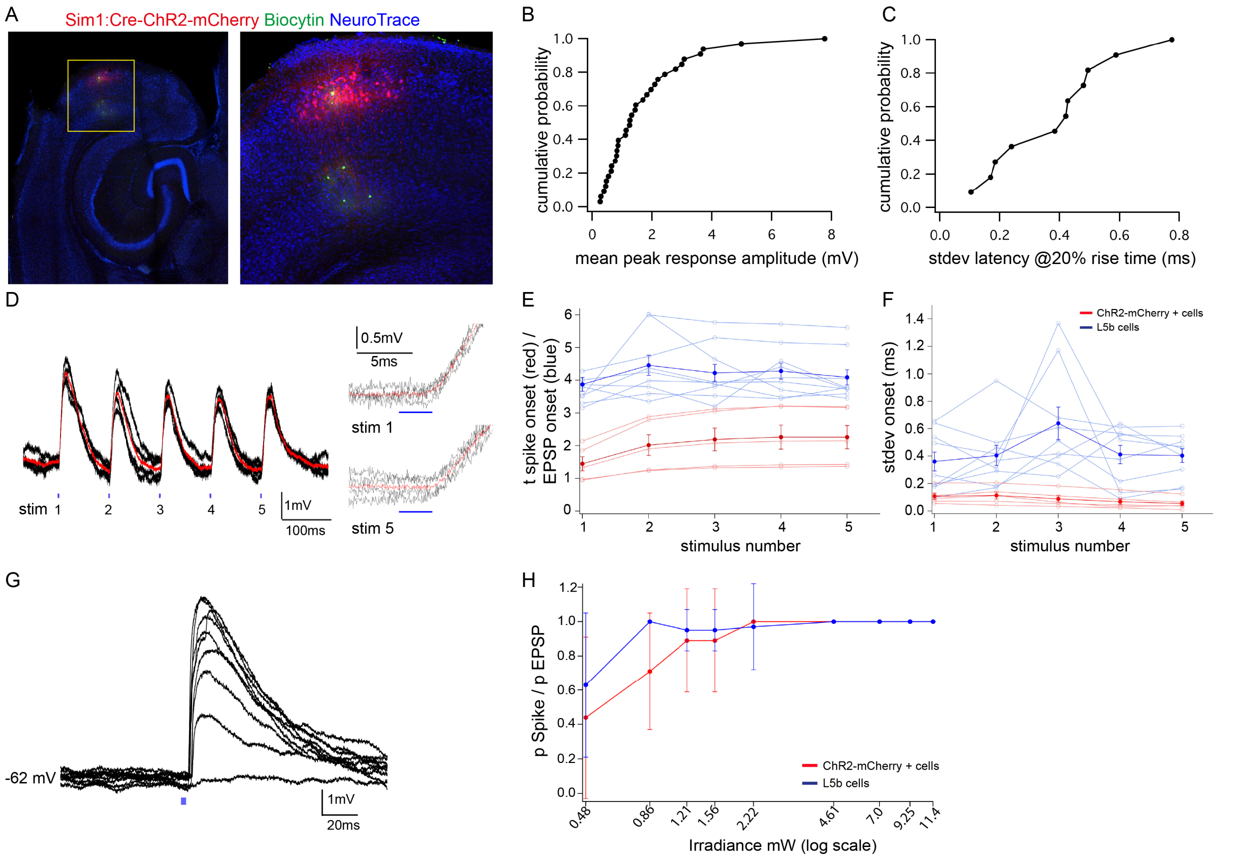

Supplemental Figure 4 (refers to Figure 4).
(A) An example horizontal slice used for patch-clamp recordings to probe connectivity between *Sim1:Cre*-ChR2-mCherry cells in Layer 2 (red) and biocytin filled deep layer neurons (green). NeuroTrace counterstaining as well as pipette positions that are imaged at the end of recordings were used as guide in mapping the layer position of recorded neurons. Experiments used either horizontal or sagittal brain slices. For both slice configurations there was a significantly higher fraction of neurons generating excitatory responses in L5b compared to L5a (horizontal p = 0, sagittal p = 0.012, Z-test). The low response probability in L5a is indistinguishable between preparations (horizontal: n = 0/23; sagittal: n =1/18; p = 0.24, Z-test), while the probability of observing excitatory responses in L5b in a sagittal slice appears to be higher than in a horizontal slice (horizontal: n = 3/12, sagittal: n = 26/39; p = 0.01, Z-test).
(B, C) Cumulative probability plots of the mean peak response amplitude (B) and standard deviation of the response latency for responding neurons in L5b (C).
(D) Examples of response to trains of stimuli at 10 Hz. Onset of the first and fifth responses are shown to the right on an expanded timescale. Time of light stimulation is indicated by the blue bars.
(E-F) The mean (E) and standard deviation (F) of the onset of spikes in L2SCs and EPSPs in L5b neurons relative to the onset of light stimulation.
(G) Examples of EPSPs recorded from an L5b neuron in response to light of increasing intensity.
(H) The probability of a spike fired by an L2SC (red) or an EPSP in an L5b neuron plotted as a function of light intensity.

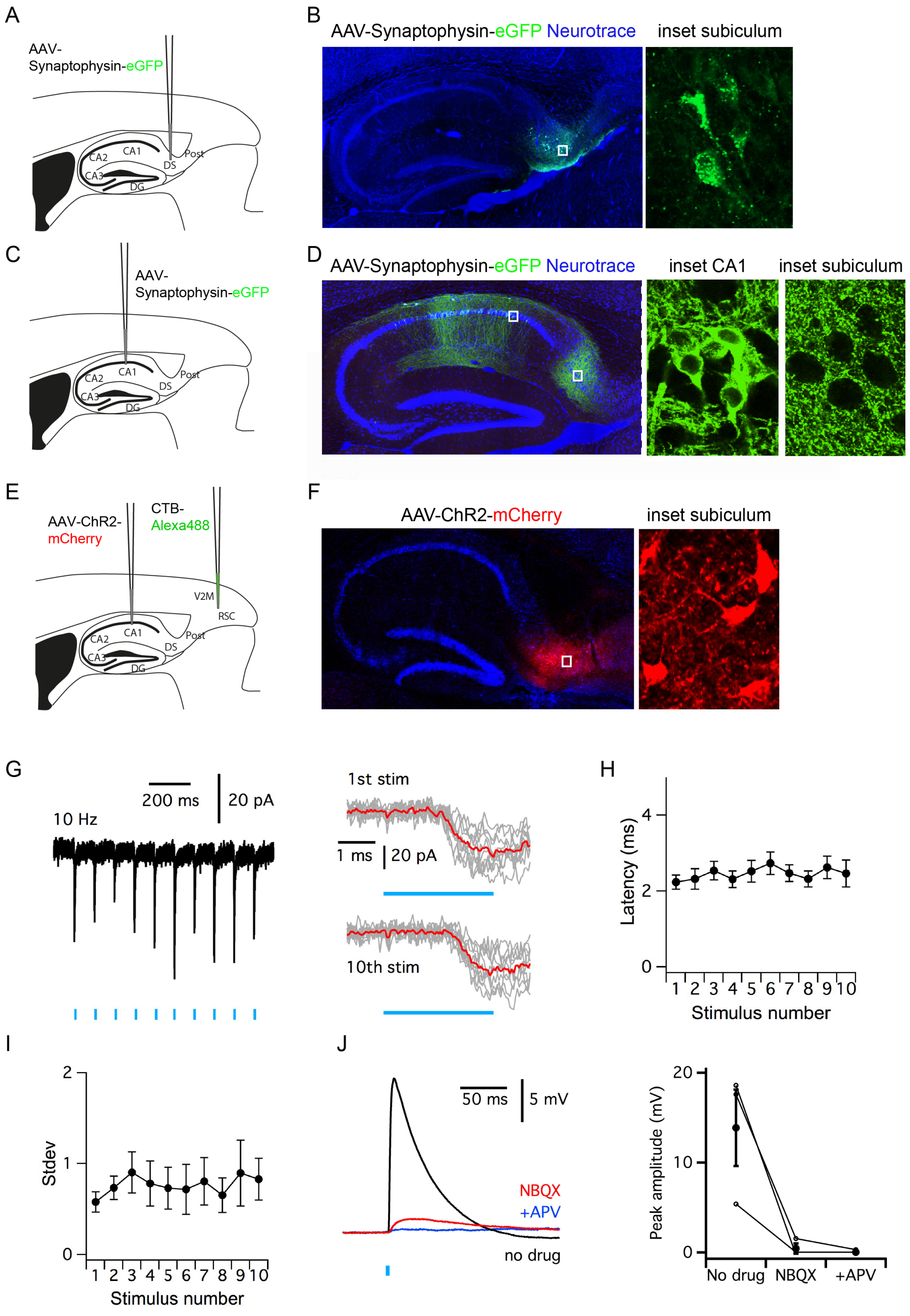

A
AAV-Synaptophysin-eGFP
CA1
CA2
CA3
DG
DS
Post
B
AAV-Synaptophysin-eGFP Neurotrace
inset subiculum
C
AAV-Synaptophysin-eGFP
D
AAV-Synaptophysin-eGFP Neurotrace
inset CA1
inset subiculum
E
AAV-ChR2-mCherry
CTB-Alexa488
V2M
RSC
F
AAV-ChR2-mCherry
inset subiculum
G
200 ms
20 pA
10 Hz
1st stim
1 ms
20 pA
10th stim
H
Latency (ms)
Stimulus number
I
Stdev
Stimulus number
J
50 ms
5 mV
NBQX
+APV
no drug
Peak amplitude (mV)
No drug
NBQX
+APV

Supplemental Figure 5 (refers to Figure 6).

(A-F) Injection strategies (A, C, E) and eGFP expression at the injection site (B, D, E) for experiments targeting injection of AAV-synaptophysin-eGFP into the dorsal subiculum (A-B) or CA1 (C-D), or AAV-ChR2-mCherry to the dorsal subiculum (E-F). Insets illustrate somatic expression of eGFP at the injection site and in (D) also show terminals of CA1 neurons in the dorsal subiculum.

(G) Examples of membrane currents in response to a 10 Hz train of 10 light pulses recorded from a neuron in L5b following expression of ChR2-mCherry in the dorsal subiculum. Insets show the onset of 1st and 10th stimuli on an expanded time scale.

(H-I) Mean latency (H) and standard deviation of the latency (I) of light-evoked responses during a 10 Hz train as a function of stimulus number.

(J) Example of mean light evoked responses of an L5b neuron to activation of inputs from the dorsal subiculum and during application of NBQX (5 µM), and then NBQX (5 µM) + D-APV (50 µM). Response amplitudes are shown to the right. Filled circles show mean responses (± SEM). Note in this experiment (J) neurons in dorsal subiculum were infected with AAV expressing the wild-type version of ChR2, whereas all other experiments use the H134R variant.
